# Challenges in the Therapy of Visceral Leishmaniasis in Brazil: A Public Health Perspective

**DOI:** 10.1155/2013/319234

**Published:** 2013-12-05

**Authors:** Edson Carvalho de Melo, Carlos Magno Castelo Branco Fortaleza

**Affiliations:** ^1^Hospital Estadual Bauru, Secretaria de Estado da Saúde de São Paulo, 17033-360 Bauru, SP, Brazil; ^2^Departamento de Doenças Tropicais e Diagnóstico por Imagem, Faculdade de Medicina de Botucatu, Universidade Estadual Paulista (UNESP), 18618-970 Botucatu, SP, Brazil

## Abstract

Over 3,000 yearly cases of Visceral Leishmaniasis (VL) are reported in Brazil. Brazilian Public Health System provides universal free access to antileishmania therapeutic options: Meglumine Antimoniate, Amphotericin B deoxycholate, and Liposomal Amphotericin B. Even though Amphotericin formulations have been advised for severe disease, this recommendation is mostly based on the opinion of experts and on analogy with studies conducted in other countries. Presently, there are two ongoing multicenter clinical trials comparing the efficacy and safety of the available therapeutic options. Some other issues require further clarification, such as severity markers and the approach to VL/AIDS coinfection. Brazil is facing the challenge of providing access to diagnosis and adequate treatment, in order to avoid VL-related deaths.

## 1. Introduction

In the second decade of the 21st century, visceral leishmaniasis (VL) continues to challenge public health authorities around the world. The World Health Organization (WHO) estimates yearly incidence of 500,000 cases, with a case-fatality ratio of approximately 10% [1, 2]. This picture is especially worrisome for the six countries that harbor 90% of VL cases: India, Bangladesh, Sudan, South Sudan, Ethiopia, and Brazil [1, 3].

*Leishmania infantum chagasi* is the agent of VL in Latin America. At least twelve Countries in the continent reported VL cases in the past decade. Brazil is by far the most affected country, with more than 3,000 reported cases per year and case-fatality ratio of 5.8% in the period from 2005 through 2009 [4].

Brazilian picture is worsened by the progressive territorial expansion and urbanization of LV. Up to the early 1980s, the disease was mainly restricted to rural areas in Northeastern Brazil [5]. In the turn of that decade, urban foci of VL were reported in major cities in that region. A southward expansion was also noticed, and the proportion of cases reported outside northeast region increased from 15% in 1998 to 44% in 2005 [6].

The changing epidemiology of visceral leishmaniasis posed new challenges for health protection of the residents of the affected areas. In the first place, there is the difficulty of prevention. Measures directed to the canine reservoirs (dog culling, vaccination) or to the vector (environmental intervention, insect repellents) are often impractical in large urban centers. Besides, they were not proved to be definitely effective [7, 8]. Another challenge concerns the management (including therapy) of VL cases. This is a relevant issue, since case-fatality ratio is generally higher in newly affected regions. São Paulo State (located in Southeastern Brazil) is an example. The first cases were reported in the State's western border in 1999. The pooled case-fatality ratio for 1999–2012 was 8.7%, and it was especially higher (11.5%) in the first five years [9]. Delay in the diagnosis and institution of appropriate therapy is a possible explanation for those data [10]. Both issues concern public health response.

The purpose of our review is to discuss the therapeutic options for VL in Brazil and public policies to ensure access throughout the country.

## 2. Therapeutic Options Available in the Brazilian VL Program

The universal and free access to health care is predicted in the Brazilian Constitution and is provided through the Unified Health System (SUS, an acronym for the Portuguese “Sistema Único de Saúde”). Presently there are three therapeutic options for VL available in the SUS: Meglumine Antimoniate (Glucantime), Amphotericin B deoxycholate, and Liposomal Amphotericin B (Ambisome).

*Pentavalent antimonials (Meglumine antimoniate and Sodium Stibogluconate)* were the first-line treatment for VL for many decades. Besides leishmanicidal activity, antimonials have been shown to induce proinflammatory responses (e.g., enhancement of phagocytosis and production of TNF-alpha), which may play a role in controlling VL progression [11–13]. Recently, the use of this class was limited in India due to extensive parasite resistance—up to 60% [14]. However, there is no evidence of significant resistance to Meglumine Antimoniate in Brazil. On the contrary, reports from observational studies point out to high efficacy. A recent study focusing on a cohort of children treated with antimonials reported efficacy of 96% in mild-to-moderate cases, and over 60% in severe cases [15]. The same study found rates above 15% for cardiac, hepatic, and pancreatic toxicity. Toxicity is indeed a major concern, and severe cases (sometimes leading to death) have been reported, mostly in patients above 50 years of age [16]. Moreover, Antimoniates are contraindicated in pregnancy and in patients with renal failure [17]. There is also concern about failures and relapses in special groups, such as those with AIDS-VL coinfection and other immune-suppressed patients [18]. The recommended dosing regimen for Glucantime is 20 mg/kg/day of Antimoniate (up to 1215 mg/day, which represent three vials), for 20–40 days.

*Amphotericin B deoxycholate (ABD)* was introduced as second-line therapy for VL in the 1960s. This drug has excellent leishmanicidal activity, with cure rates of 90%–95% in studies from endemic countries [19]. Similar to antimonials, ABD (as well as lipid formulations) was also shown to have immune modulatory effects, including regulation of T-cell proliferation and enhancement of pro-inflammatory cytokines production [20–22].

There are, however, significant obstacles to the widespread use of this drug. The administration must be slow in order to avoid infusion-related adverse effects. This fact makes the treatment in an inpatient or day-hospital scheme necessary. Furthermore, the nephrotoxicity rates may be higher than 50% [23]. Until 2013, Brazilian guidelines for treatment of severe VL recommended the choice of Amphotericin B deoxycholate for patients in extremes of age (over 65 years or under 6 months), with signs of malnourishment or significant comorbidities. The recommended posology is 1 mg/Kg/day (maximum 50 mg/day) for 14–21 days [24]. A recent document from Brazilian Health Ministry (September 2013) updated that recommendation [25]. In the revised guidelines, the groups cited above should be preferably treated with Liposomal Amphotericin.

The lipid formulations of Amphotericin B greatly increased the safety in the therapy of systemic mycosis and VL [26]. Presently there are three formulations in the market: Amphotericin lipid complex (Abelcet), Amphotericin colloidal dispersion (Amphocil), and *Liposomal Amphotericin (AmBisome).* Though they are not bioequivalent (and probably not therapeutically equivalent), all of them allow the administration of doses above 5 mg/kg/day. Current data suggest that liposomal amphotericin is less toxic [26]. This formulation is also the only one that has been extensively studied in the therapy of VL [27].

Several dosing schemes of Liposomal Amphotericin have been studied for VL in different countries, ranging from single doses of 10 mg/kg [28] to a total of 18–21 mg/kg/day distributed over 5–7 days (i.e., 3–5 mg/kg/day) [29]. The reported efficacy is generally above 90% [30].

In Brazil, recommended doses are 3-4 mg/kg/day for 5–7 days (Ministry of Health) [18, 24, 25] and 3–5 mg/kg/day for 5 days (São Paulo State Health Department) [31].

The major limitation for the use of Liposomal Amphotericin is its price, and this fact has weighed negatively on cost-effectiveness analysis [32]. However, this has been overcome by an agreement with the producing industry (Gilead), which allowed Brazilian Health Ministry to purchase AmBisome at prices much lower than those prevailing in the market.

## 3. Therapeutic Challenges: Severe Patients and AIDS Coinfection

The Brazilian Health Ministry recommendations for severe VL favor the use of liposomal Amphotericin B. The evidence for this choice is not strong. It is based on the opinion of experts and on analogy from clinical trials mostly conducted in India [33]. While the urgent need for reducing case-fatality rates justifies decisions based on incomplete evidence, some major questions remain. What is the definition of “severe VL”? Which are the clinical signs and laboratory markers of severity?

Four recent studies focusing on predictors of death in VL patients showed agreement in most findings [10, 34–36]. Briefly, delay in the diagnosis, age above 60 years, hemorrhagic manifestations, jaundice, suspected or confirmed bacterial infection, neutropenia, low platelet counts, and AIDS coinfection were associated with worse outcomes. All those factors, alongside with anemia, renal failure, malnourishment, and low serum albumin (<2.5 mg/dL), are listed as severity markers in the most recent governmental recommendations for treatment of severe VL [24, 25]. Although this document does not indicate unequivocally the use of amphotericin B (deoxycholate or liposomal) for patients in this condition, it determines the availability and logistics to supply these drugs throughout the country. Given the country continental extent and the disparities in social/economic development among regions—which have an impact on patients access to health services—this commitment is of foremost importance.

AIDS/VL coinfection is also an important issue in Brazil. Trends in VL reemergence (urbanization) and AIDS epidemiology (interiorization, i.e., progress towards cities in inner Brazil) made covered areas of the two diseases intertwine [37]. Thus, similar to what had happened in Europe and Africa [38], co-infection became epidemiologically and clinically relevant. In 2007-2008, approximately 4% of VL cases in Brazil were coinfections [39]. This proportion varied among States and reached nearly 10% in São Paulo.

A comprehensive account of specificities of AIDS/VL co-infection is beyond the objectives of this review. Still, some of the major challenges are worth citing: (a) atypical and severe presentations, which hinder diagnosis and impact unfavorably on outcomes [40, 41]; (b) failure of therapy with antimonials [18]; and (c) frequent relapses and the need for long-term suppressive therapy [42]. Given the unfavorable outcomes of co-infected patients, Brazilian Health Ministry revised therapeutic guidelines in year 2013, including HIV co-infection as an indication for therapy with Liposomal Amphotericin.

The beneficial effect of highly active antiretroviral therapy (HAART) in the prevention of symptomatic VL or of relapses is still not clear, both in Brazil or in a worldwide perspective [42]. This is an important issue, since patients with VL/AIDS co-infection may benefit from the universal access to antiretrovirals provided by the Brazilian Aids Program [43].

## 4. The São Paulo State Experience

At this point, making a brief description of the governmental approach of VL therapy in the State of São Paulo is worth. This is the most populous and economically developed among the 26 states in Brazil. As discussed earlier, the first autochthonous case of VL was reported in 1999. In the first five years, pooled case-fatality ratio was 11.5%—about two times the value for Brazil. In an effort to lower death rates, the State expedited guidelines in 2006, recommending therapy with Liposomal Amphotericin for all children up to 10 years of age and adults over 50 years. VL/AIDS co-infection, pregnancy, and relapses were also defined as indications for Liposomal Amphotericin [31]. Those recommendations were considerably wider than those proposed by the Brazilian Health Ministry at that time [17]. In years that followed the expedition of the State Guideline (2006–2012), the case-fatality ratio was 7.8% (RR = 0.67; 95% CI, 0.50–0.91, *P* = 0.01). Of course, many issues may account for this finding (including improvement in diagnosis and access to health system). Still, a more refined analysis of the Database for Notifiable Diseases may throw some light on the possible beneficial effect of the change in São Paulo State therapeutic recommendations.

## 5. Difficulties and Future Prospects

Some of the challenges for the treatment of visceral leishmaniasis in Brazil are generic. They relate to the difficulties of providing free health care throughout the territory of a huge developing country. It is worth remembering that, especially in the Amazon Region, VL patients may have difficulty in transportation to centers where treatment is available. In this sense, the Public Health System has sought empowerment of regional centers for the care of VL cases.

Other challenges are more specific. Little is known about the effectiveness of treatment options available in SUS. We emphasized previously how much the current recommendations are based on expert opinion and the analogy with clinical trials that focused on Indian or African VL. Here, too, the Ministry of Health of Brazil has undertaken significant effort. Through its department of science and technology (DECIT) and the Brazilian Innovation Agency (FINEP), two multicenter clinical trials were funded, with the aim of comparing the efficacy and safety of the available therapeutic options. One of those trials is also assessing combination therapy with different drugs, a strategy that proved useful in other countries [29, 44]. When available, data from those trials will provide valuable data to guide definite governmental recommendations.

Since the effect of preventive strategies is not established [8], timely diagnosis and adequate therapy may be the most important goals to avoid VL-related deaths. The development and validation of a unified severity score could help in guiding therapeutic choice. A thorough approach to data from Epidemiologic Surveillance Files may also be of help in providing information on the effectiveness of VL therapy in a “real-life” setting. Those are important issues for research. While many questions remain on the efficacy and cost-effectiveness of VL therapeutic options in Brazil, it is certain that the country is making great progress toward an evidence-based approach to this potentially life-threatening disease.
